# The Care of the Feeble-Minded at Leicester

**Published:** 1910-11-26

**Authors:** 


### THE CARE OF THE FEEBLE-MINDED AT LEICESTER.

A conference on this subject, at which Mr. J. Howe (chairman of the Leicester Guardians) presided, was called at Leicester by that body to consider the question of the future care and control of epileptic and feeble-minded persons chargeable to the rates. Mr. T. Cope (Chairman of the County Council) and the Hon. Gerald Walsh (Local Government Board Inspector) were present, as well as representatives from every Board of Guardians in the country.

Mr. J. L. Harrison (Leicester Guardians) read a paper advocating that the Boards of Guardians should follow the excellent example set by Birmingham, Aston, and King's Norton Board and combine for the special purpose of providing more efficiently for the mentally defective under their care. This combination could be brought about at once, without waiting for legislation such as would be necessary to establish a new authority; and if the Government did eventually decide to establish such an authority for dealing with the mentally defective, it would not be difficult to transfer the colony now proposed.

The Chairman of the County Council hoped the Boards would give the matter their earnest consideration; and Dr. Clifton (Chairman of the Borough Lunatic Asylum) and others approved of the proposal.

The following resolution was then carried: "That in order to provide more efficiently for the care and control of the mentally defective, this Conference is of opinion that the Leicestershire and Rutland Boards of Guardians, together with the Borough Board of Guardians, should form a joint committee for this purpose." One representative from each Union was chosen to form a provisional committee to prepare a scheme. Mr. H. Mansfield (Clerk to the Borough Guardians) was appointed clerk for the time being to this committee.

